# Supplementary material for: Co-Occurrence of Domestic Dogs and Gastropod Molluscs in Public Dog-Walking Spaces and Implications for Infection with Angiostrongylus vasorum: A Preliminary Study
Source: Animals (Basel). 2021 Sep 2;11(9):2577. doi: 10.3390/ani11092577 (PMC8472295; doi:10.3390/ani11092577)
Supplement: Supplementary file 1 [file animals-11-02577-s001.zip › animals-1292551-supplementary.pdf]

Model term	Pairwise comparison	Likelihood ratio parameter estimate	*SE*	*z*	*p*
Habitat	Natural grassland vs hardstanding	2.844	0.439	6.481	«0.001***
	Natural grassland vs amenity grassland	2.082	0.278	7.480	«0.001***
	Woodland/scrub vs amenity grassland	2.766	0.382	7.251	«0.001***
	Woodland/scrub vs hardstanding	3.528	0.511	6.911	«0.001***

SI Table S1. Parameter estimates and pairwise comparisons for factors predicting gastropod presence/absence (0,1) across 7 study sites in Brighton and Hove and surrounding areas

Model term	Pairwise comparison	Likelihood ratio parameter estimate	*SE*	*z*	*p*
Habitat	Woodland/scrub vs natural grassland	0.443	0.231	1.922	< 0.05*
	Woodland/scrub vs amenity grassland	0.498	0.290	1.721	0.055·
	Woodland/scrub vs hardstanding	0.870	0.522	1.669	0.065·

SI Table S2. Parameter estimates and pairwise comparisons for factors predicting gastropod relative abundance across 7 study sites in Brighton and Hove and surrounding areas. · approaches significance.

Model term	Pairwise comparison	Likelihood ratio parameter estimate	*SE*	*z*	*p*
Habitat	Natural grassland vs hardstanding	2.470	0.631	3.193	«0.001***
	Natural grassland vs amenity grassland	1.741	0.340	5.119	«0.001***
	Woodland/scrub vs amenity grassland	0.283	0.415	7.919	«0.001***
	Woodland/scrub vs hardstanding	4.013	0.681	5.893	«0.001***
	Woodland/scrub vs Natural grassland	1.543	0.348	4.431	«0.001***
Minimum relative humidity (%)	Continuous	-2.068	0.597	-3.465	< 0.001***
Time of day	PM vs AM	1.113	0.335	3.319	< 0.001***
	PM vs DAY	0.884	0.321	2.759	< 0.01**

SI Table S3. Parameter estimates and pairwise comparisons for factors predicting presence/absence of *Arion ater* and *hortensis* aggregates, and *Cornu aspersum* (taxa harbouring medium and high prevalence of *Angiostrongylus vasorum* from the literature) across 7 study sites in Brighton and Hove and surrounding areas.

Model term	Pairwise comparison	Likelihood ratio parameter estimate	*SE*	*z*	*p*
Time of day	AM vs PM	1.892	0.286	6.627	«0.001***
	MID vs PM	1.886	0.300	6.291	«0.001***
Habitat	Hardstanding vs Natural grassland	1.306	0.346	3.771	<0.001***
	Hardstanding vs Woodland/scrub	2.078	0.358	5.804	«0.001***
	Amenity grassland vs Natural grassland	0.622	0.281	2.215	<0.05*
	Amenity grassland vs Woodland/scrub	1.393	0.283	4.922	«0.001***
	Natural grassland vs woodland/scrub	0.771	0.272	2.831	<0.01**
Footpath	On footpath versus off footpath	0.481	0.227	2.124	<0.05*
Minimum relative humidity (%) [continuous]		-0.016	0.009	-1.878	0.060·

SI Table S4. Parameter estimates and pairwise comparisons for significant effects predicting dog presence/absence (0,1) across 7 study sites in Brighton and Hove and surrounding areas. AM = 06.30-10.30; MID (midday) = 12.30-16.30; PM = 18.30-22.30. · approaches significance.

**Model term**	**Pairwise comparison**	**Likelihood ratio parameter estimate**	***SE***	***z***	***p***
Time of day	AM vs PM	0.730	0.174	4.210	« 0.001***
	PM vs MID	-0.615	0.176	-3.502	< 0.001***
Habitat	Amenity grassland vs Natural grassland	0.642	0.123	5.210	« 0.001***
	Hardstanding vs Natural grassland	0.497	0.133	3.727	< 0.001***
	Woodland/scrub vs Natural grassland	0.712	0.118	6.011	« 0.001***
Footpath	On footpath versus off footpath	0.514	0.256	2.011	<0.05*
Minimum relative humidity (%) [continuous]		-0.012	0.003	-3.935	« 0.001***

SI Table S5. Parameter estimates and pairwise comparisons for significant effects predicting dog (*Canis lupus familiaris*) abundance across 7 study sites in Brighton and Hove and surrounding areas. AM = 06.30-10.30; MID (midday) = 12.30-16.30; PM = 18.30-22.30. · approaches significance.
